# Chicken and Duck Myotubes Are Highly Susceptible and Permissive to Influenza Virus Infection

**DOI:** 10.1128/JVI.03421-14

**Published:** 2014-12-24

**Authors:** Belinda Baquero-Perez, Suresh V. Kuchipudi, Jemima Ho, Sujith Sebastian, Anita Puranik, Wendy Howard, Sharon M. Brookes, Ian H. Brown, Kin-Chow Chang

**Affiliations:** a School of Veterinary Medicine and Science, University of Nottingham, Loughborough, Leicestershire, United Kingdom; b Virology Department, Animal and Plant Health Agency, Weybridge, Addlestone, Surrey, United Kingdom

## Abstract

Skeletal muscle, at 30 to 40% of body mass, is the most abundant soft tissue in the body. Besides its primary function in movement and posture, skeletal muscle is a significant innate immune organ with the capacity to produce cytokines and chemokines and respond to proinflammatory cytokines. Little is known about the role of skeletal muscle during systemic influenza A virus infection in any host and particularly avian species. Here we used primary chicken and duck multinucleated myotubes to examine their susceptibility and innate immune response to influenza virus infections. Both chicken and duck myotubes expressed avian and human sialic acid receptors and were readily susceptible to low-pathogenicity (H2N3 A/mallard duck/England/7277/06) and high-pathogenicity (H5N1 A/turkey/England/50-92/91 and H5N1 A/turkey/Turkey/1/05) avian and human H1N1 (A/USSR/77) influenza viruses. Both avian host species produced comparable levels of progeny H5N1 A/turkey/Turkey/1/05 virus. Notably, the rapid accumulation of viral nucleoprotein and matrix (M) gene RNA in chicken and duck myotubes was accompanied by extensive cytopathic damage with marked myotube apoptosis (widespread microscopic blebs, caspase 3/7 activation, and annexin V binding at the plasma membrane). Infected chicken myotubes produced significantly higher levels of proinflammatory cytokines than did the corresponding duck cells. Additionally, in chicken myotubes infected with H5N1 viruses, the induction of interferon beta (IFN-β) and IFN-inducible genes, including the melanoma differentiation-associated protein 5 (MDA-5) gene, was relatively weak compared to infection with the corresponding H2N3 virus. Our findings highlight that avian skeletal muscle fibers are capable of productive influenza virus replication and are a potential tissue source of infection.

**IMPORTANCE** Infection with high-pathogenicity H5N1 viruses in ducks is often asymptomatic, and skeletal muscle from such birds could be a source of infection of humans and animals. Little is known about the ability of influenza A viruses to replicate in avian skeletal muscle fibers. We show here that cultured chicken and duck myotubes were highly susceptible to infection with both low- and high-pathogenicity avian influenza viruses. Infected myotubes of both avian species displayed rapid virus accumulation, apoptosis, and extensive cellular damage. Our results indicate that avian skeletal muscle fibers of chicken and duck could be significant contributors to progeny production of highly pathogenic H5N1 viruses.

## INTRODUCTION

In 1997, the first case of highly pathogenic avian influenza (HPAI) H5N1 virus transmission from poultry to humans was documented in Hong Kong (1). Despite extensive culling of poultry at the time, genetically related Eurasian HPAI H5N1 viruses emerged some years later (2), and since 2005, they have become panzootic in domestic poultry and wild birds in countries in three different continents (3–5). Classical Eurasian HPAI H5N1 viruses circulating before the contemporary Eurasian HPAI H5N1 viruses produced few or no clinical signs in ducks (6–9). While some strains of contemporary Eurasian HPAI H5N1 viruses are able to kill juvenile ducks (10, 11), other strains remain nonlethal to ducks (12, 13). Epidemiologically, domestic ducks are believed to contribute to the maintenance of HPAI H5N1 viruses in eastern Asia (14, 15) and to be sources of outbreaks in susceptible poultry (16, 17). In contrast, classical and contemporary Eurasian HPAI H5N1 viruses in chickens are highly lethal, killing up to 100% of the animals within a few days of infection (13, 18).

The striking contrast in clinical outcomes between ducks and chickens observed within days of infection suggests that there are host species-specific differences in innate immunity. There are few reported studies that compare the responses of different avian host species to the same avian influenza virus infection *in vitro* (19–21), in part due to the limited methodology available for the isolation of different types of primary avian cells. Primary avian skeletal muscle cells are a significant cell type for studying the avian host response to influenza virus infection for several reasons. (i) Although there is mostly no viral spread to skeletal muscle in chickens infected with low-pathogenicity avian influenza (LPAI) viruses (22, 23), with HPAI H5N1 viruses, recovery of viral proteins, RNA, and, more importantly, live virus from muscle of experimentally infected chickens (23–25) and of naturally and experimentally infected ducks has been reported (10, 26, 27). (ii) Skeletal muscle is the largest soft tissue type in poultry. Combined, deboned thigh, deboned drumstick, and breast meat represent 35% and 16% of the total live weight of adult broiler chickens and ducks, respectively (Cherry Valley Farms Ltd., personal communication). Skeletal muscle therefore represents a major site for virus deposition following systemic spread of the virus (viremia). (iii) Epidemiologically, skeletal muscle can play a role in the spread of avian influenza viruses. Chickens fed HPAI H5N1 virus-contaminated chicken meat succumbed to rapid infection and death (23). Moreover, meat from apparently healthy ducks served as a direct route of infection of HPAI H5N1 virus to humans who handle meat as well as those who consume it (25, 27). (iv) Muscle is considered a major innate immune tissue/organ. It has the capacity to produce cytokines and chemokines (28, 29) and respond to proinflammatory cytokines (30). (v) Lastly, skeletal myofibers (myotubes) are different from other cell types in that they are postmitotic multinucleated cells wherein hundreds of nuclei share a syncytium, and importantly, unlike many other RNA viruses, the influenza virus genome replicates in the nucleus of infected cells rather than in the cytoplasm. The rich concentration of nuclei within a fiber syncytium could conceivably facilitate virus replication, leading to increased virion assembly and release from infected cells. However, to date, the dynamics of influenza virus replication in postmitotic multinucleated muscle fibers are poorly understood. We report here a comparative study of the host innate immune responses of primary chicken and duck skeletal muscle cells to influenza virus infection. We found that both chicken and duck myotubes are highly susceptible to avian influenza virus infections and subsequently undergo extensive apoptosis. Additionally, there are significant the differences in the host innate responses between myotubes of chicken and those of duck, consistent with their relative susceptibility to influenza virus infection.

## MATERIALS AND METHODS

### Primary chicken and duck muscle cells and MDCK cells.

Primary muscle satellite cells from 4- to 6-week-old Pekin ducks (Anas platyrhynchos), from Cherry Valley Farms UK, and 4- to 6-week-old broiler chickens (Gallus gallus) (ROSS 308 strain), from P. D. Hook Hatcheries, Thirsk, United Kingdom, were isolated as previously described (31). All animals were euthanized by the administration of pentobarbital in accordance with Schedule 1 of the Animals (Scientific Procedures) Act of 1986. Muscle satellite cells were grown as myoblasts and differentiated into multinucleated myotubes by using Dulbecco's modified Eagle's medium (DMEM)-Glutamax I (high glucose) (Invitrogen) with 10% horse serum, 4% chicken embryo extract (Egg Technologies), and 1% penicillin-streptomycin (P/S). For each avian species, primary muscle satellite cells were pooled from several animals for infection studies. The culture flasks used were coated with 1% type I collagen from rat tail (Sigma) diluted in sterile water. Following isolation from pectoral muscles, all cells were subjected to a maximum of two passages (trypsinization), after which (unfused) myoblasts were frozen in 90% horse serum (HS) and 10% dimethyl sulfoxide and stored in liquid nitrogen. For experiments requiring myotubes, myoblasts were seeded and allowed to proliferate and differentiate in culture for several days for maximal fusion into myotubes. Approximately 65% of avian muscle cells routinely fused into postmitotic myotubes (31). Madin-Darby canine kidney (MDCK) cells (ATCC CCL-34) were cultured by using DMEM-Glutamax I (high glucose) with 10% fetal calf serum and 1% P/S.

### Virus infection and detection.

A LPAI H2N3 virus (A/mallard duck/England/7277/06); two HPAI H5N1 viruses, A/turkey/England/50-92/91 and A/turkey/Turkey/1/05, referred to as HPAI H5N1 50-92 and HPAI H5N1 tyTy05, respectively; and a human H1N1 (A/USSR/77) virus were used in this study. HPAI H5N1 50-92 virus is from the “classical” Eurasian lineage of H5 viruses and causes high rates of mortality in chickens but no mortality in ducks (9). HPAI H5N1 tyTy05 virus, a contemporary Eurasian H5N1 virus (clade 2.2) associated with the global panzootic outbreak, can cause deaths in juvenile ducks (10). All viruses were grown by allantoic inoculation of 10-day-old embryonated chicken eggs. All HPAI H5N1 virus infection work was carried out in the Advisory Committee on Dangerous Pathogens biological containment level 3 (ACDP CL3)/Specified Animal Pathogens Order 1998 containment level 4 (SAPO4) facility at the Animal and Plant Health Agency, Weybridge.

Myoblasts or myotubes were washed once with phosphate-buffered saline (PBS) and infected in serum-free infection medium (DMEM–F-12; Invitrogen) supplemented with 2% Ultroser G (Pall Corporation), 1% P/S, 1% insulin-transferrin-selenium (Invitrogen), and 500 ng/ml l-1-tosylamide-2-phenylethyl chloromethyl ketone (TPCK) trypsin. After 2 h of virus incubation at the specified multiplicity of infection (MOI), based on virus titration (focus-forming assay) on MDCK cells by immunocytochemical detection of viral NP protein at 6 h of infection, the infection medium was removed, and cells were carefully washed twice with warm PBS, followed by replenishment with fresh infection medium for a further period of incubation as specified in each experiment.

Cells were processed and immunolabeled by using an EnVision+ system-HRP (DAB) kit (Dako), as previously described (20, 31). Briefly, cells were fixed in acetone-methanol for 10 min, followed by a 10-min incubation with peroxidase block solution and incubation for 40 min with a mouse monoclonal (AA5H) antibody against viral nucleoprotein (Abcam) at 1 μg/ml. The cells were then rinsed with Tris-buffered saline (TBS) and incubated with a horseradish peroxidase-labeled polymer for 40 min. After further rinsing with TBS, the cells were incubated with DAB substrate for 6 min, washed with TBS, and counterstained with Harris' hematoxylin.

Additionally, viruses were titrated in MDCK cells to determine the median tissue culture infectious dose (TCID_50_) (32). In brief, supernatants were used to infect MDCK cell monolayers in 96-well plates. MDCK cells were washed free of serum present in the maintenance medium by using serum-free DMEM. Half-log dilutions of the supernatants were made across the plate, using four rows per sample. The virus was allowed to adsorb for 1 h at 37°C before the inoculum was removed and replaced with DMEM supplemented with l-glutamine and P/S. Plates were incubated for 3 days at 37°C with 5% CO_2_. Supernatants were then removed, and the presence of replicating virus was identified by using crystal violet (Sigma) staining to identify intact cell monolayers (no virus) versus nonintact cell monolayers (virus present). TCID_50_ values per ml of supernatant were calculated by using the Spearman-Kärber formula (33).

### Metabolic and caspase 3/7 assays.

Primary chicken and duck muscle cells were seeded onto 96-well culture plates (∼5,000 cells per well) and cultured for several days until myotubes were well formed. The metabolic activity of muscle cells at 24 h postinfection (p.i.) was determined by using CellTiter 96 (Promega), a nonradioactive cell proliferation assay which is a modification of the 3-(4,5-dimethylthiazol-2-yl)-2,5-diphenyltetrazolium bromide (MTT) reduction assay. Activated caspases 3 and 7 in muscle cells were quantified at different times of infection by using a Caspase-Glo 3/7 assay kit (Promega).

### Annexin V and propidium iodide detection.

Phosphatidylserine (PS) translocation and membrane integrity were monitored with the use of an annexin V-enhanced green fluorescent protein (EGFP) apoptosis detection kit (Source Bioscience). Live cells (not fixed) were incubated for 5 min in the dark with annexin V-EGFP and propidium iodide (PI), followed by visualization with a fluorescence microscope (DFC490; Leica).

### Quantitative real-time PCR.

Total RNA was isolated from cells grown in 12-well plates by using an RNeasy Plus minikit (Qiagen). Cells were homogenized with a QIAshredder homogenizer (Qiagen), followed by the removal of genomic DNA (gDNA) using a gDNA eliminator column. Each cDNA synthesis reaction was performed with 1 μg of total RNA by using a SuperScript III first-strand synthesis kit (Invitrogen).

Real-time PCR was performed by using SYBR green I and TaqMan chemistries. Primers and TaqMan probes for chicken and duck interferon alpha (IFN-α), chicken and duck interleukin 6 (IL-6), chicken and duck interleukin 8 (IL-8), and duck tumor necrosis factor alpha (TNF-α) were designed with Primer Express version 2.0 (Applied Biosystems), based on previously reported sequences (Table 1). Owing to the absence of a conventionally recognized TNF-α gene sequence in chicken, a lipopolysaccharide-induced TNF-α factor (LITAF) gene sequence was used in its place. The primers and probe for the chicken 2′,5′-oligoadenylate synthetase (*2*′,*5*′-OAS) gene (A, B, and like forms) were designed with the Roche online Universal Probe Library (UPL) Assay Design Center. Primers for chicken IFN-β, described previously (34), were used with SYBR green I chemistry. Primers and probes for the chicken Mx1 gene, the chicken protein kinase R (PKR) gene, and duck retinoic acid-inducible gene 1 (RIG-I) (35); chicken melanoma differentiation-associated gene 5 (MDA-5) (36); the viral M gene (37); and the chicken 18S rRNA gene (38) were synthesized as previously described. The conditions for quantitative PCR (qPCR) using TaqMan and UPL probes were 10 min at 95°C followed by 45 cycles of 95°C for 15 s, 60°C for 1 min, and 72°C for 1 s, followed by cooling for 10 s at 4°C. The cycling program for qPCR using SYBR green I was a 5-min preincubation step at 95°C followed by 45 cycles of 95°C for 30 s, 60°C for 30 s, and 72°C for 10 s. At the end of the PCR cycles, a melting curve analysis was performed to ascertain a single amplification product. Each cDNA sample was amplified in triplicate, and the mean values were calculated for each gene. mRNA levels were normalized to the 18S rRNA gene and expressed as fold changes relative to mock-infected cells at each p.i. time point. The mean fold change for each gene was determined from three biological replicates. The sequences and probes used in this study are presented in Table 1.

**TABLE 1:** Sequences of primers and probes used for quantitative real-time PCR

Gene	Primer or probe^*a*^	Sequence (5′→3′) or description	GenBank accession no.
18S rRNA	F	TGTGCCGCTAGAGGTGAAATT	AF173612.1
	R	TGGCAAATGCTTTCGCTTT	
	P	TTGGACCGGCGCAAGACGAAC	
Chicken TNF-α-like factor (LITAF)	F	CCCTTCTGAGGCATTTGGAA	AY765397
	R	CAGCCTGCAAATTTTGTCTTCTT	
	P	AGCCCACTCCCGAACGCTG	
Chicken IFN-α	F	CTTCCTCCAAGACAACGATTACAG	EU367971
	R	AGGAACCAGGCACGAGCTT	
	P	CCTGCGCCTGGGAACACGTCC	
Chicken IL-6	F	CACGATCCGGCAGATGGT	EU170468
	R	TGGGCGGCCGAGTCT	
	P	ATAAATCCCGATGAAGTGGTCATCC	
Chicken 2′,5′-OAS	F	GGTGCTCTTCATCAACTGCTT	NM_204609.1
	R	CTCGATGATGGCGAGGAT	
	P	UPL probe15	
Chicken IL-8	F	CCCTCGCCACAGAACCAA	NM_205018.1
	R	CAGCCTTGCCCATCATCTTT	
	P	CCCAGGTGACACCCGGAAGAAACA	
Duck TNF-α	F	GCCAACAAATAACCCCGTTACA	EU375296
	R	CTGGGCGGTCATAAAATACCA	
	P	CAGGTTGCTGCACATACACCGTCTGAA	
Duck IFN-α	F	AACCAGCTTCAGCACCACATC	DQ861429
	R	TGTGGTTCTGGAGGAAGTGTTG	
	P	TGCTTCCCAGCCGACGCC	
Duck IL-6	F	CCAAGGTGACGGAGGAAGAC	AB191038
	R	TGGAGAGTTTCTTCAAGCATTTCTC	
	P	TGTCTCCTGGCTGGCTTCGACGA	
Duck IL-8	F	AGCCTGGTAAGGATGGGAAAC	AB236334.1
	R	GGGTGGATGAACTTCGAGTGA	
	P	AGCTCCGGTGCCAGTGCATAAGCA	

a F, forward primer; R, reverse primer; P, probe.

### One-step quantitative reverse transcription-PCR of viral RNA in culture supernatants.

Viral RNA from culture supernatants was extracted by using a High Pure viral RNA kit (Roche). A one-step reverse transcription-PCR (RT-PCR) assay using influenza virus M gene-specific primers and probe was performed as previously described (37, 39). Threshold cycle (*C_p_*) values were converted to viral gene copy numbers by a standard curve generated using *in vitro*-transcribed viral RNA. In order to take into account residual virus inoculum after the PBS washes, supernatants were collected from each well (0-h time point), and M gene RNA was quantified by RT-PCR. The signal obtained at 0 h was then subtracted from the corresponding 12- and 24-h signals. Supernatants collected from the two time points were made from two series of culture wells. Reverse transcription for cDNA synthesis was performed at 50°C for 15 min. PCR amplification consisted of an initial preincubation step at 95°C for 2 min followed by 40 cycles of 95°C for 15 s, 60°C for 30 s, and 72°C for 1 s.

### Lectin cytochemistry.

Chicken and duck muscle cells were grown on 24-well Nunclon flat-bottom plates (Nunc), as they did not proliferate well on glass coverslips. Lectin labeling was performed as previously described (40). Cells were fixed with 4% paraformaldehyde in PBS for 10 min at room temperature. Endogenous biotin activity was blocked by the use of a streptavidin/biotin blocking kit (Vector Laboratories). Cells were then incubated overnight at 4°C in the dark with fluorescein isothiocyanate (FITC)-labeled Sambucus nigra agglutinin (SNA) lectin (human α-2,6-linked sialic acid receptor binding) and biotinylated Maackia amurensis agglutinin II (MAA II) lectin (avian α-2,3-linked sialic acid receptor binding) (Vector Laboratories), both at 10 μg/ml. Subsequently, the cells were washed three times with TBS and incubated for 2 h at room temperature with a streptavidin-Alexa Fluor 594 conjugate (Invitrogen). Cells were washed again three times with TBS and mounted with ProLong Gold antifade reagent with 4′,6′ diamidino-2-phenylindole (DAPI) (Invitrogen). Negative controls were processed without the use of lectin.

## RESULTS

### Influenza virus-infected chicken and duck myotubes but not myoblasts progressively accumulate viral NP.

**FIG 1:**
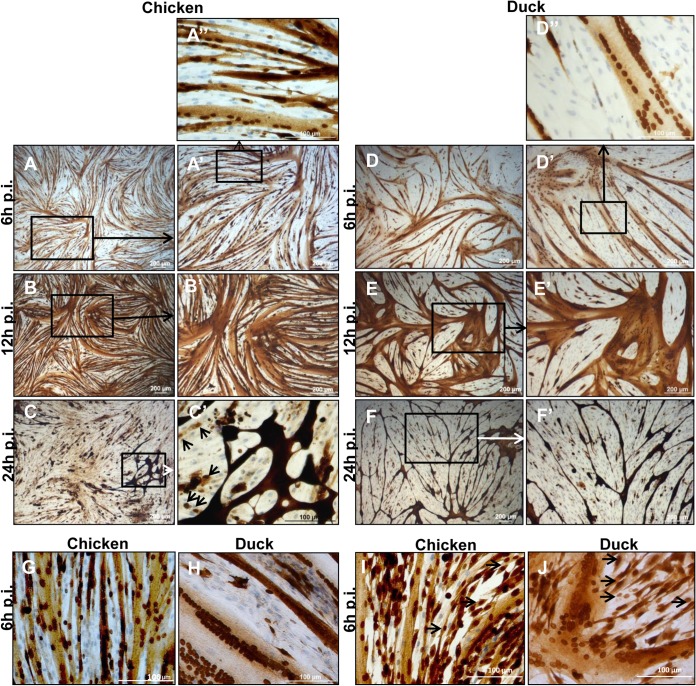
Infected chicken and duck myotubes but not myoblasts show progressive accumulation of viral NP. (A to A″ and D to D′) At 6 h p.i. with LPAI H2N3 virus at a MOI of 0.1, almost all chicken and duck myotubes but only a few myoblasts were immunopositive for viral NP (brown). (B, B′, E, and E′) At 12 h p.i., more viral NP accumulated in chicken and duck myotubes, as evident by the intensity of NP detection. (C, C′, F, and F′) By 24 h p.i., the remaining attached chicken and duck myotubes showed even more intense NP expression, while few myoblasts were infected (C′, arrows). All cells were immunolabeled at the same time; differences in the intensity of labeling indicate different amounts of intracellular NP. Cells were counterstained with Harris' hematoxylin to visualize nuclei. (G and H) A similar NP expression outcome with human H1N1 (A/USSR/77) virus at a MOI of 0.1 was found for chicken and duck myotubes (data from 6 h p.i. are shown). (I and J) At a higher MOI of 1.0 with LPAI H2N3 virus, chicken and duck myotubes and myoblasts (arrows) showed comparable viral NP expression levels (data from 6 h p.i. are shown).

We recently reported a simple but effective method for the isolation of muscle satellite cells from several avian and mammalian species, including chicken and duck (31). Differentiated chicken and duck myotubes were infected with a LPAI H2N3 virus at a MOI of 0.1. Chicken and duck myotube cultures were spatially monitored for viral NP by immunocytochemistry at 6 h, 12 h, and 24 h p.i. (Fig. 1). At 6 h p.i., NP was detected in virtually all myotubes, but only a small proportion of myoblasts appeared to be infected. NP strongly accumulated in the nuclei and, to a lesser degree, in the sarcoplasm of infected chicken (Fig. 1A and A′) and duck (Fig. 1D to D″) myotubes. At 12 h p.i., viral NP became more intense in the sarcoplasm of chicken (Fig. 1B and B′) and duck (Fig. 1E and E′) myotubes, but infected nuclei remained strongly labeled for NP. By 24 h p.i., widespread myotube detachment from most of the culture surface had occurred. The remaining chicken (Fig. 1C and C′) and duck (Fig. 1F and F′) myotubes appeared to accumulate even more viral NP. The difference in infection patterns between myotubes and myoblasts of both avian species was also seen with the human H1N1 virus (A/USSR/77) at a MOI of 0.1 (Fig. 1G and H, respectively). Myoblasts, however, were not completely resistant to influenza virus infection; with LPAI H2N3 virus at a MOI of 1.0, both myotubes and myoblasts from chicken and duck were comparably labeled for viral NP (Fig. 1I and J, respectively). In summary, avian myotubes appeared highly susceptible to influenza virus infection.

### Infected myotubes show greater accumulation of virus M gene RNA than do the corresponding myoblasts or MDCK cells.

**FIG 2:**
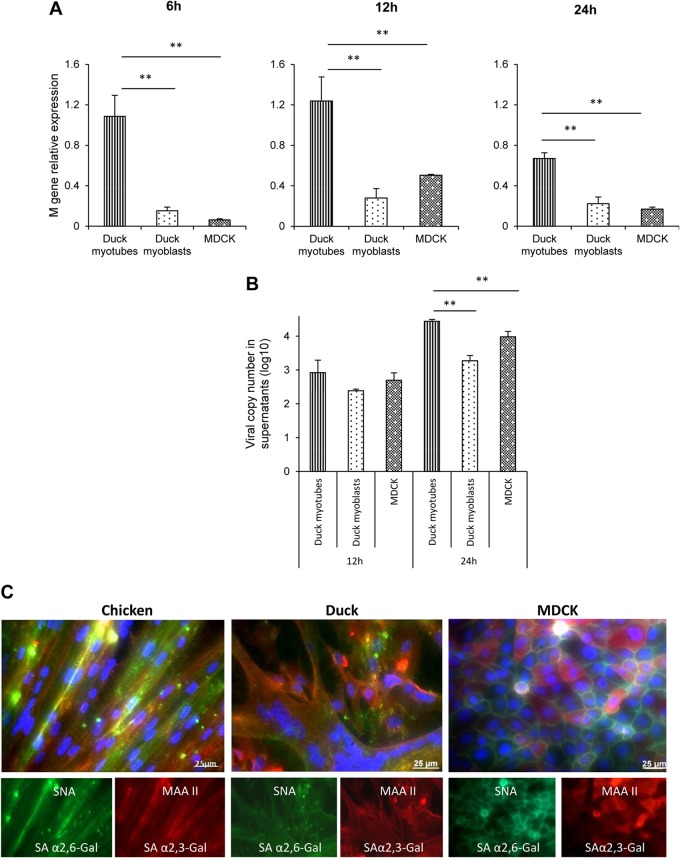
Infected duck myotubes show higher levels of accumulation of virus M gene RNA than do the corresponding myoblasts or MDCK cells. (A) Duck myotube, duck myoblast, and MDCK cell cultures were infected with LPAI H2N3 virus at a MOI of 0.1. Duck myotubes had significantly (*P* < 0.005) higher levels of intracellular viral M gene RNA (normalized to the 18S RNA gene) than did myoblasts and MDCK cells at 6 h, 12 h, and 24 h of infection. (B) Duck myotubes also accumulated the most viral M gene RNA in culture supernatants by 24 h of infection. Results show the means of data from three biological replicates, with error bars indicating standard deviations. One-way analysis of variance followed by Tukey's multiple-comparison test was used (**, *P* < 0.005). (C) Chicken and duck muscle cells (myotubes and myoblasts) and MDCK cells coexpressed avian and human sialic acid receptor types. The human α-2,6-linked sialic acid receptor (green) and avian α-2,3-linked sialic acid receptor (red) were detected with Sambucus nigra agglutinin (SNA) and Maackia amurensis agglutinin II (MAA II) lectins, respectively. Nuclei were counterstained by using DAPI (blue). Merged and individual fluorescent images show extensive expression of both receptors in all three cell types.

The intracellular and extracellular accumulation of viral M gene RNA was determined in duck myotube, duck myoblast, and MDCK cell cultures infected with LPAI H2N3 virus at a MOI of 0.1. At 6 h, 12 h, and 24 h p.i., duck myotubes produced significantly higher levels(*P* < 0.005) of intracellular viral M gene RNA than did similarly infected myoblasts and MDCK cells (Fig. 2A). Similar results were obtained with the corresponding chicken myotubes (data not shown). Viral M gene RNA in culture supernatants of duck myotubes at 24 h p.i., as assessed by one-step reverse transcription real-time PCR, was significantly more abundant than in the other two cell types (Fig. 2B). Taken together, these results suggest that the viral replication rate was higher in avian myotubes than in mononuclear cells. Since myoblasts and myotubes from both avian species as well as MDCK cells expressed both the human and avian influenza receptor types (α-2,6-linked sialic acid and α-2,3-linked sialic acid, respectively) (Fig. 2C), it is likely that the observed relatively high viral expression levels of M gene RNA and NP protein in myotubes were not due to differences in the early stages of virus receptor binding and virus entry.

### Avian influenza virus-infected chicken and duck myotubes show comparable progeny virus outputs and similar reductions in cell viability.

**FIG 3:**
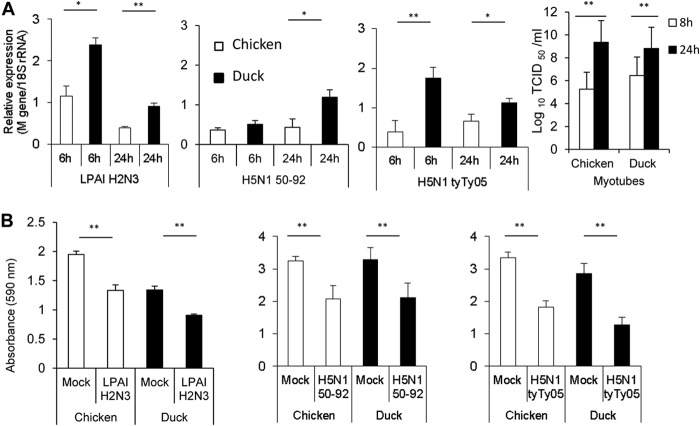
Avian influenza virus-infected chicken and duck myotubes show comparable progeny virus outputs and similar reductions in cell viability. (A) One LPAI H2N3 and two HPAI H5N1 viruses at a MOI of 1.0 conferred higher levels of accumulation of M gene RNA in duck than in chicken myotubes (*, *P* < 0.05; **, *P* < 0.005 [determined by an unpaired *t* test]); however, comparable increasing outputs of progeny H5N1 tyTy05 virus were detected for both avian species, based on TCID_50_ virus assays using infected supernatants on MDCK cells (*, *P* < 0.05 [determined by a two-sample *t* test]). (B) Chicken and duck myotubes infected at a MOI of 1.0 for 24 h displayed a significant reduction in cell viability based on MTT assays (**, *P* < 0.005 [determined by an unpaired *t* test]). There was no significant difference in reduced viability between infected chicken and infected duck myotubes. Data points are the means of data from four wells of a 96-well plate, with error bars indicating standard deviations.

Chicken and duck myotubes were infected with three different avian influenza viruses (LPAI H2N3, HPAI H5N1 50-92, and HPAI H5N1 tyTy05) at a MOI of 1.0 over a period of 24 h. Viral M gene RNA accumulation, normalized to 18S rRNA, in duck myotubes was consistently higher than that in the corresponding chicken myotubes for all three viruses (Fig. 3A); a similar relative difference in M gene expression was also found for influenza virus-infected duck and chicken primary fibroblasts (our unpublished data). However, both chicken and duck myotubes infected with H5N1 tyTy05 virus at a MOI of 1.0 produced comparable increasing levels of progeny virus from 8 to 24 h of infection (Fig. 3A). MTT assays to determine the resulting metabolic rates (cell viability) at 24 h p.i. showed similar reductions in infected chicken and infected duck myotubes (Fig. 3B). Therefore, infected chicken and duck myotubes showed similar reductions in cell viability and were comparably permissive to the production of viable H5N1 tyTy05 progeny virus.

### Influenza virus-infected chicken and duck myotubes show extensive cytopathic damage.

**FIG 4:**
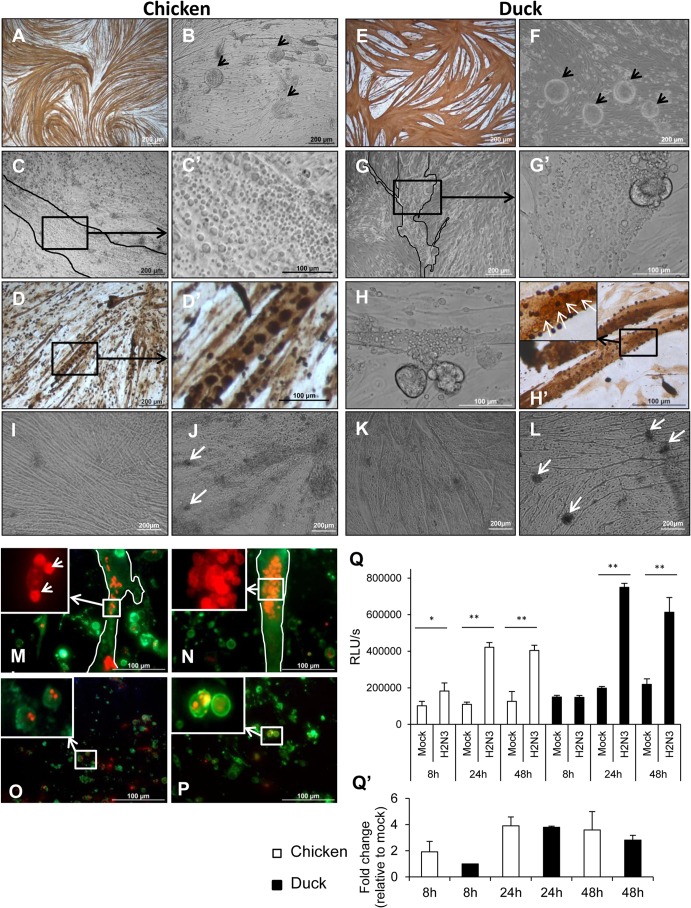
Influenza virus-infected chicken and duck myotubes show extensive cytopathic damage. Chicken and duck myotubes were infected with LPAI H2N3 virus at a MOI of 1.0 for 24 h. (A and E) Uninfected control chicken and duck myotubes show extensive expression of the muscle-specific intermediate filament desmin (brown). (B and F) Cytopathic damage of chicken and duck myotubes is seen as widespread rounding and detachment of cells (arrows). (C, C′, G, and G′) Whole chicken and duck myotubes appear to have degenerated into numerous small membrane-lined blebs (apoptotic bodies) (myotube boundaries are delineated). (D, D′, H, and H′) Commonly, sections of chicken and duck myotubes contained aggregates of apoptotic blebs with concentrated viral NP, as evident by immunocytochemical NP detection. Morphologically intact myonuclei were found in the sarcoplasm of the duck myotube (H′, arrows). (I and K) Phase-contrast microscopy of mock-infected chicken (I) and duck (K) myotube cultures show typical myotubes. (J and L) In contrast, similar myotube detachment and rounding (arrows) were evident in HPAI H5N1 50-92 virus-infected (MOI of 1.0) chicken (J) and duck (L) myotubes at 24 h p.i. (M to P) Translocation of PS to the outer membrane was detected (green fluorescence) by annexin V-EGFP binding to chicken and duck muscle cells (myotubes and myoblasts) infected with LPAI H2N3 virus (MOI of 1.0) for 24 h. Infected chicken (M) and duck (N) myotubes were extensively positive for PS translocation; some cells had lost membrane integrity, as evident by the nuclear uptake of PI (red fluorescence). Myonuclei labeled with PI displayed chromatin condensation and/or fragmentation in chicken and duck myotubes, whose boundaries are outlined (M and N, respectively). A morphologically intact nucleus with condensed and/or fragmented nuclear contents is highlighted (M, arrows). Chromatin condensation and fragmentation were also seen in chicken (O) and duck (P) myoblasts. (Q) Chicken and duck myotube cultures infected with LPAI H2N3 virus at a MOI of 1.0 significantly activated the effector caspases 3 and 7 at 24 h and 48 h p.i. (*, *P* < 0.05; **, *P* < 0.01 [determined by an unpaired *t* test]) (RLU/s, relative light units per second). (Q′) Results are also represented as fold changes. There was, however, no significant difference in the activation of caspases 3 and 7 between the two avian species. Data points are the means of data from three wells from a 96-well plate, with error bars indicating standard deviations.

Differentiated chicken and duck myotubes were infected with a LPAI H2N3 virus at a MOI of 1.0. Uninfected control chicken and duck cultures typically displayed extensive swirls of myotubes immunopositive for muscle-specific intermediate desmin filaments (Fig. 4A and E, respectively). At 24 h p.i., severe cytopathic damage was evident, with widespread rounding or detachment of chicken and duck myotubes (Fig. 4B and F, respectively). There were numerous sarcoplasmic blebs, which appeared as small membrane-lined vesicles associated with degenerating myotubes (Fig. 4C to D′ and G to H′, respectively). These blebs appeared to be apoptotic bodies, a feature well recognized in other cell types but not previously observed in myotubes undergoing apoptosis *in vitro* (41, 42). Concentrations of viral NP colocalized with sarcoplasmic blebs in both infected chicken and duck myotubes (Fig. 4D and D′ and H and H′, respectively). Similar cytopathic changes were observed in chicken and duck myotubes infected with HPAI H5N1 50-92 virus (Fig. 4J and L, respectively). To further examine the cytopathic changes in chicken and duck myotubes infected with LPAI H2N3 virus at a MOI of 1.0 for 24 h, PS membrane translocation (apoptotic change) was localized by annexin V-EGFP binding, visualized as green fluorescence (Fig. 4M to P). Some annexin V-EGFP-positive cells had lost their membrane integrity, as demonstrated by the uptake of the red nuclear fluorochrome PI, indicating late apoptosis or even necrosis. PI-labeled myonuclei showed chromatin condensation and/or fragmentation in chicken and duck myotubes (Fig. 4M and N, respectively). Similar apoptotic changes were also detected in infected chicken and duck myoblasts (Fig. 4O and P, respectively). Both chicken and duck myotube cultures displayed similar and significant activation of caspases 3 and 7 at 24 h and 48 h after infection (Fig. 4Q and Q′). In summary, extensive and severe chicken and duck myotube damage from LPAI and HPAI virus infections was accompanied by clear hallmarks of apoptosis and evidence of necrosis.

### Chicken myotubes display more vigorous cytokine responses to avian influenza virus infection than do duck myotubes.

**FIG 5:**
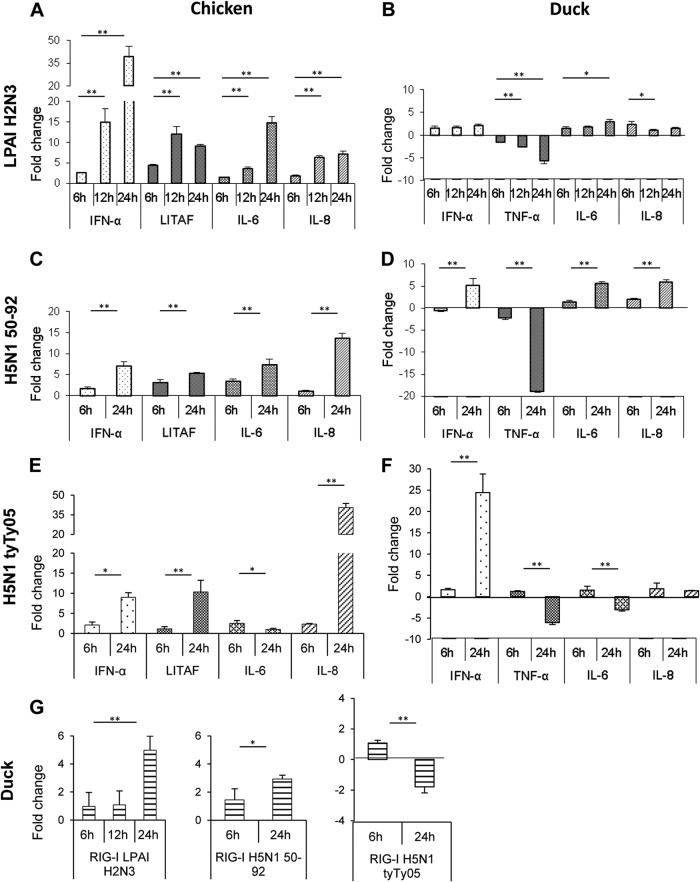
Chicken myotubes display a more vigorous cytokine response to avian influenza virus infection than do duck myotubes. (A and B) Chicken myotubes infected with a LPAI H2N3 virus showed a strong induction of IFN-α (40-fold induction) and a moderate induction of proinflammatory cytokines (LITAF, IL-6, and IL-8) (A); the corresponding duck muscle cells showed weaker induction, with progressive downregulation of the TNF-α response (B). (C and E) With HPAI H5N1 50-92 (C) or HPAI H5N1 tyTy05 (E) virus, the level of IFN-α mRNA induction was not >10-fold, while IL-8 mRNA was more strongly induced in chicken than in duck muscle cells. (D and F) In duck cells, TNF-α gene transcription was downregulated by both HPAI H5N1 viruses, unlike LITAF gene induction in the corresponding chicken cells (C and E). Duck IFN-α mRNA induction was greater with HPAI H5N1 tyTy05 virus (F) than with HPAI H5N1 50-92 virus (D). (G) Duck myotubes infected with LPAI H2N3 and HPAI H5N1 50-92 viruses showed modest upregulation of the viral RNA sensor RIG-I, but with HPAI H5N1 tyTy05 virus, RIG-I expression was downregulated. mRNA levels were normalized to the 18S rRNA gene and are expressed as fold changes in relation to uninfected controls at each p.i. time point. The fold change for each gene is the mean of data from three biological replicates, with error bars indicating standard deviations. A significant increase or decrease in mRNA levels between 6 h p.i. and later times of infection was determined by a two-sample unpaired *t* test (*, *P* < 0.05; **, *P* < 0.01).

Following the findings of extensive cell death associated with prominent apoptotic changes in infected chicken and duck myotubes, we examined the expression of IFN-α and the proinflammatory cytokines TNF-α, IL-6, and IL-8 in these infected cells (MOI of 1.0 over 24 h). The IFN-α gene transcript was strongly upregulated (≈40-fold increase) in differentiated chicken muscle cells at 24 h p.i. with LPAI H2N3 virus (Fig. 5A). Additionally, levels of the proinflammatory cytokines LITAF (TNF-α), IL-6, and IL-8 were clearly upregulated at 12 h and 24 h p.i. in chicken muscle cells. In contrast, the corresponding duck muscle cells displayed only a modest induction of IFN-α, IL-6, and IL-8 and a progressive downregulation of TNF-α (Fig. 5B). Next, chicken myotubes were infected with HPAI H5N1 50-92 virus at an MOI of 1.0. Unexpectedly, this HPAI virus elicited a less vigorous cytokine response than did the LPAI H2N3 virus; in particular, the level of IFN-α induction was <10-fold at 24 h p.i. (Fig. 5C). In duck myotubes, HPAI H5N1 50-92 virus infection also induced a weak cytokine response, with a notable downregulation of TNF-α at 24 h p.i. (Fig. 5D). Chicken myotubes infected with HPAI H5N1 tyTy05 virus did not exceed 10-fold IFN-α induction at 24 h p.i. (Fig. 5E). Among the four proinflammatory cytokines, IL-8 was most strongly induced (40-fold increase). Duck myotubes infected with the same high-pathogenicity virus displayed a weak proinflammatory response, with downregulation of TNF-α and IL-6 at 24 h of infection. Duck IFN-α showed a 25-fold induction (Fig. 5F). On the whole, chicken myotubes appeared to be more proinflammatory in response to LPAI and HPAI viruses than did the corresponding duck myotubes.

We further examined the expression of duck RIG-I, a key cytoplasmic pattern recognition receptor (PRR) believed to play a key role in the innate resistance of ducks but which is absent in chickens (35). Infection of duck myotubes with LPAI H2N3 and HPAI H5N1 50-92 viruses, which do not usually cause clinical disease in ducks, resulted in a small induction of RIG-I. HPAI H5N1 tyTy05 virus infection in duck muscle cells, which *in vivo* is lethal to juvenile ducks (10), downregulated RIG-I expression (Fig. 5G).

### LPAI H2N3 virus induces a more vigorous antiviral response than do HPAI H5N1 viruses in chicken myotubes.

**FIG 6:**
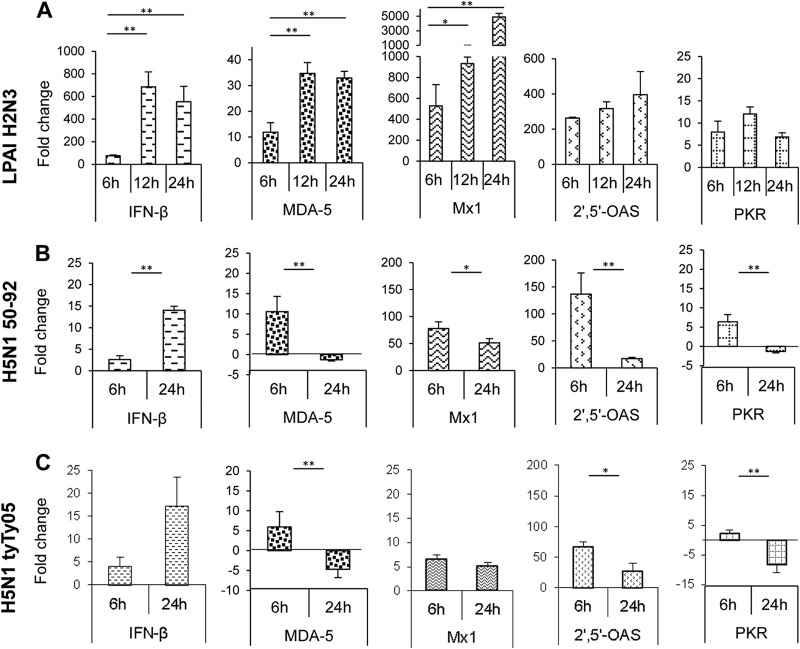
LPAI H2N3 virus induces a more vigorous antiviral response than do HPAI H5N1 viruses in chicken myotubes. (A) Chicken myotubes infected with LPAI H2N3 virus at a MOI of 1.0 induced a strong upregulation of IFN-β, which correlated with the upregulation of the viral RNA sensor MDA-5 as well as the IFN-inducible Mx1, 2′,5′-OAS, and PKR genes. (B and C) However, with HPAI H5N1 50-92 (B) and HPAI H5N1 tyTy05 (C) viruses, both at a MOI of 1.0, the level of IFN-β mRNA induction was <20-fold, considerably lower than that for the corresponding LPAI H2N3 virus infection (A). In contrast to LPAI H2N3 virus infection (A), HPAI H5N1 50-92 (B) and HPAI H5N1 tyTy05 (C) viruses at 24 h p.i. downregulated the expression of the MDA-5 and PKR genes and elicited much weaker induction of Mx1 and 2′,5′-OAS gene expression. mRNA levels were normalized to the 18S rRNA gene and are expressed as fold changes in relation to uninfected controls at each p.i. time point. The fold change for each gene is the mean of data from three biological replicates, with error bars indicating standard deviations. A significant increase or decrease in mRNA levels between 6 h p.i. and later times of infection was calculated by a two-sample unpaired *t* test (*, *P* < 0.05; **, *P* < 0.01).

We next focused on the expression of the chicken IFN-β gene and three interferon-inducible genes, Mx1, 2′,5′-OAS, and PKR, which are all known to have antiviral activity against influenza A virus. We also quantified the expression level of MDA-5, a previously identified PRR of influenza virus in chicken cells (43). Chicken myotubes at 6 h p.i. with LPAI H2N3 (MOI of 1.0) showed a 75-fold induction of IFN-β transcription (Fig. 6A). The level of production of IFN-β peaked at 12 h p.i. (≈685-fold increase) and remained high at 24 h p.i. (≈555-fold). IFN-β induction correlated with the upregulation of the MDA-5, Mx1, 2′,5-OAS, and PKR genes. Notably, the Mx1 gene was highly upregulated during early infection. Mx1 gene expression showed ≈530-fold, ≈930-fold, and ≈5,000-fold increases at 6 h, 12 h, and 24 h p.i., respectively. The 2′,5′-OAS gene was also strongly upregulated, reaching an ≈400-fold increase at 24 h of infection. In contrast, chicken cells infected with HPAI H5N1 50-92 (Fig. 6B) and HPAI H5N1 tyTy05 (Fig. 6C) viruses (both at a MOI of 1.0), showed relatively modest IFN-β induction, with a <20-fold increase. Lower-level IFN-β induction was accompanied by the downregulation of the MDA-5 and PKR genes and much weaker Mx1 and 2′,5′-OAS gene responses at 24 h p.i. than with the corresponding LPAI H2N3 virus infection. Collectively, chicken myotubes appeared to mount a more robust antiviral response to LPAI H2N3 virus than to HPAI H5N1 viruses.

## DISCUSSION

### Chicken and duck myotubes are highly susceptible to avian influenza virus infection and subsequent cellular damage.

Not much is known about the role of skeletal muscle in the pathogenesis of influenza virus infection in poultry. It is particularly important to evaluate the permissiveness of duck skeletal muscle to productive influenza virus infection since such an avian species is highly resistant to the development of clinical disease, making duck muscle (meat) a potential source of human public health threats and a mode for disease spread between birds in the wild. We found that chicken and duck myotubes were highly susceptible to LPAI H2N3 virus infection, even at a relative low MOI of 0.1 (Fig. 1 and 2). There was progressive accumulation of viral NP in chicken and duck myotubes, accompanied by levels of intracellular and extracellular viral M gene RNA that were higher than those in the corresponding myoblasts and MDCK cells. Viral NP was extensively detected inside cultured myotubes as early as 6 h p.i., which was consistent with the rapid detection and progressive buildup of HPAI H5N1 virus in skeletal muscles from 6 h after intranasal inoculation into chickens (24). Both chicken and duck myotubes infected with H5N1 tyTy05 virus at a MOI of 1.0 showed a progressive increase of progeny virus output from 8 to 24 h of infection (Fig. 3A). Indeed, viable HPAI H5N1 virus had been isolated from contaminated duck meat exported to South Korea from China (25). Myotubes but not myoblasts have been reported to support influenza virus replication by hemadsorption assays (44). The apparent high rate of viral activity in myotubes may well be related to the abundance of nuclei in the sarcoplasmic syncytium, which could readily receive invading viral RNPs (vRNPs), uncoated from endosomes, to initiate primary transcription and subsequent viral genome replication. It was demonstrated previously that *de novo* vRNPs with associated M1 protein assembled in the nucleus and transported to the cytosol are not able to reenter the nucleus, based on an analysis of interspecies heterokaryons containing nuclei from infected and uninfected cells (45).

The other distinctive feature of influenza virus-infected myotubes was the dramatic manifestations of cellular damage. Chicken and duck myotubes infected with LPAI and HPAI H5N1 viruses showed comparable reductions in cell viability (Fig. 3B). Likewise, LPAI H2N3 virus infection (at a MOI of 1.0 for 24 h) of chicken and duck myotubes resulted in widespread myotube loss and degeneration accompanied by the accumulation of microscopic blebs, caspase 3/7 activation, and annexin V binding at the plasma membrane, all of which were indicators of apoptosis (Fig. 4). However, necrotic changes could not be ruled out due to the extent of cellular damage. Similar cytopathic changes were seen with the use of the human H1N1 (A/USSR/77) virus (data not shown). Skeletal muscle cells are known to highly express endogenous caspase inhibitors (46, 47); however, avian influenza viruses appeared to be able to effectively induce extensive apoptosis. Our findings are consistent with observations made previously by Desdouits et al. (48), who found that human myotubes are much more susceptible to infection with pandemic and seasonal influenza H1N1 viruses than are the corresponding myoblasts, resulting in cytopathic damage and a greater yield of progeny viruses.

### Innate response of chicken and duck myotubes to avian influenza virus infection.

We evaluated the innate responses of chicken and duck myotubes to LPAI H2N3 and HPAI H5N1 virus infection. In the main, chicken myotubes displayed more vigorous proinflammatory responses to LPAI and HPAI H5N1 viruses than did the corresponding duck cells (Fig. 5A to F). Notably, the TNF-α gene transcript was downregulated in duck myotubes with all three viruses; in contrast, chicken myotubes showed an upregulation of LITAF, a TNF-α like factor. TNF-α mediates strong endothelial activation and clotting activity (49), which would contribute to the pathological damage found in HPAI virus-infected chickens (18). We also found a similarly reduced proinflammatory response to H5N1 tyTy05 virus infection in primary duck lung cells relative to chicken lung cells (our unpublished data). The attenuated proinflammatory response in duck cells is not directly related to reduced virus replication, as H5N1 tyTy05 virus replicates to high titers in ducks, with detectable viral shedding from the oropharynx and cloaca (50). It is likely that attenuated proinflammation is an inherent feature of the response of ducks to H5N1 tyTy05 virus infection.

Infection of chicken myotubes with LPAI H2N3 virus resulted in potent induction of IFN-β mRNA at 12 h p.i. (≈685-fold increase), which correlated with the upregulation of the interferon-inducible MDA-5, Mx1, 2′,5′-OAS, and PKR genes (Fig. 6A). MDA-5, an RNA virus sensor, is critical for the mediation of IFN-β production in chicken cells during influenza virus infection (43). Strikingly, chicken Mx1 RNA levels reached an almost 5,000-fold increase at 24 h of infection. It was reported previously that the antiviral activity of the Mx1 protein is dependent on the amino acid asparagine (Asn) at position 631 and that serine at the same position (Ser631) rendered the protein with no antiviral activity (51). However, chicken Mx1 protein (with Asn631) conferred no apparent antiviral activity against A/WSN/33 H1N1 influenza virus in chicken embryo fibroblasts derived from three commercial breeds of chickens (52). In contrast, chickens homozygous for the Asn631 allele were significantly more resistant to HPAI virus infection (53). The functionality of chicken Mx1 therefore remains incompletely understood.

Both HPAI H5N1 viruses used in this study did not induce IFN-α and IFN-β responses in chicken myotubes as strongly as those induced by the LPAI virus (Fig. 5C and E and 6B and C). The HPAI H5N1 50-92 virus has an alanine residue at position 149 in its NS1 protein similar to the A/goose/Guangdong/1/96 H5N1 virus, where this amino acid makeup was shown to be critical for antagonizing type I IFN production in chicken embryo fibroblasts (54). Furthermore, an alanine residue at position 144 in the NS1 protein of the A/chicken/Yamaguchi/7/04 H5N1 virus was also shown to be required to counteract the type I IFN response in chicken HD-11 macrophages and chicken DF-1 fibroblasts (43, 55). Interestingly, the NS1 protein of HPAI H5N1 tyTy05 virus carries an alanine residue at position 144, and it was previously found that infection of embryonated chicken eggs with this virus did not elicit type I IFN protein production at 24 h p.i. (55). Both HPAI H5N1 viruses downregulated the MDA-5 and PKR genes at 24 h p.i. and weakly induced Mx1 and 2′,5′-OAS gene expression relative to that induced by LPAI H2N3 virus (Fig. 6B and C). Our findings reinforce the notion that HPAI H5N1 viruses are more able to inhibit type I IFN responses in certain cell types, including avian myotubes, than are LPAI viruses (55, 56).

The finding that chicken skeletal muscle cells were able to mount a vigorous antiviral response to LPAI virus infection but failed to respond in a similar manner following HPAI H5N1 virus infection indicates that HPAI H5N1 viruses (probably through the inhibition of MDA-5 via NS1 mediation) are able to effectively suppress a major innate antiviral mechanism present in chicken skeletal muscle cells. Considering that IFN-β has been suggested to have a protective role during influenza virus infection that cannot be compensated for by IFN-α (57), limited IFN-β production during H5N1 virus infection would largely reduce the antiviral capacity in chicken muscle. The impaired antiviral response elicited by HPAI H5N1 viruses *in vitro* is consistent with the *in vivo* finding of high levels of HPAI H5N1 virus in skeletal muscle of infected chickens (23–25). High levels of infectious virus and viral M gene RNA have also been reported for skeletal muscle of turkeys infected experimentally with HPAI H7N1 virus (58). The more limited annotated sequence data for the duck genome meant that we were not able to investigate the antiviral response of duck cells to the same extent as for chicken cells. Nonetheless, we demonstrated that infected duck myotubes showed a much reduced proinflammatory response to LPAI and HPAI H5N1 virus infection relative to that of chicken cells. In duck myotubes, RIG-I, a viral RNA sensor of influenza virus induced by type I IFN (59), was modestly upregulated by LPAI H2N3 and HPAI H5N1 50-92 virus infection but downregulated by HPAI H5N1 tyTy05 virus, a strain that is lethal to young ducks (10) (Fig. 5G). Our observed pattern of RIG-I induction is consistent with the role of RIG-I, thought to be absent in chickens, in mediating a crucial antiviral IFN-β response in ducks (35). However, given that chicken and duck myotubes were comparably susceptible to infection with LPAI and HPAI H5N1 viruses, the expression of RIG-I alone in duck myotubes appeared to be insufficient to resist virus replication.

In summary, our results highlight the possibility that in chickens and ducks, following systemic spread of certain HPAI H5N1 virus strains, skeletal muscle may serve as a major amplification site for virus replication. The ability of avian skeletal muscle cells to generate cytokines, which are released directly into the bloodstream, could conceivably contribute to the pathogenesis of the disease. The presence of live HPAI H5N1 virus in skeletal muscle of chickens and ducks poses a significant risk of infection for humans who handle sick or dead poultry. Contaminated poultry meat/products fed to other birds or mammals without prior cooking can also aid in the spread of HPAI H5N1 viruses.

## References

[1] Chan PKS 2002 Outbreak of avian influenza A(H5N1) virus infection in Hong Kong in 1997. Clin Infect Dis 34:S58–S64. doi:10.1086/338820. 11938498

[2] Guan Y, Peiris JSM, Lipatov AS, Ellis TM, Dyrting KC, Krauss S, Zhang LJ, Webster RG, Shortridge KF 2002 Emergence of multiple genotypes of H5N1 avian influenza viruses in Hong Kong SAR. Proc Natl Acad Sci U S A 99:8950–8955. doi:10.1073/pnas.132268999. 12077307 PMC124404

[3] Peiris JSM, de Jong MD, Guan Y 2007 Avian influenza virus (H5N1): a threat to human health. Clin Microbiol Rev 20:243–267. doi:10.1128/CMR.00037-06. 17428885 PMC1865597

[4] Guan Y, Smith GJD, Webby R, Webster RG 2009 Molecular epidemiology of H5N1 avian influenza. Rev Sci Tech 28:39–47. 19618617 10.20506/rst.28.1.1868

[5] Sims LS, Brown IH 2008 Multi-continental epizootics of H5N1 highly pathogenic avian influenza 1996–2007, p 251–286 *In* Swayne DE (ed), Avian influenza. Blackwell Publishing, Oxford, United Kingdom.

[6] Perkins LEL, Swayne DE 2003 Comparative susceptibility of selected avian and mammalian species to a Hong Kong-origin H5N1 high-pathogenicity avian influenza virus. Avian Dis 47:956–967. doi:10.1637/0005-2086-47.s3.956. 14575094

[7] Kishida N, Sakoda Y, Isoda N, Matsuda K, Eto M, Sunaga Y, Umemura T, Kida H 2005 Pathogenicity of H5 influenza viruses for ducks. Arch Virol 150:1383–1392. doi:10.1007/s00705-004-0473-x. 15747052

[8] Alexander DJ, Parsons G, Manvell RJ 1986 Experimental assessment of the pathogenicity of eight avian influenza A viruses of H5 subtype for chickens, turkeys, ducks and quail. Avian Pathol 15:647–662. doi:10.1080/03079458608436328. 18766567

[9] Wood GW, Parsons G, Alexander DJ 1995 Replication of influenza-A viruses of high and low pathogenicity for chickens at different sites in chickens and ducks following intranasal inoculation. Avian Pathol 24:545–551. doi:10.1080/03079459508419093. 18645810

[10] Londt B, Nunez A, Banks J, Nili H, Johnson LK, Alexander DJ 2008 Pathogenesis of highly pathogenic avian influenza A/turkey/Turkey/1/2005 H5N1 in Pekin ducks (Anas platyrhynchos) infected experimentally. Avian Pathol 37:619–627. doi:10.1080/03079450802499126. 19023759

[11] Kim JK, Seiler P, Forrest HL, Khalenkov AM, Franks J, Kumar M, Karesh WB, Gilbert M, Sodnomdarjaa R, Douangngeun B, Govorkova EA, Webster RG 2008 Pathogenicity and vaccine efficacy of different clades of Asian H5N1 avian influenza A viruses in domestic ducks. J Virol 82:11374–11382. doi:10.1128/JVI.01176-08. 18786988 PMC2573247

[12] Brown JD, Stallknecht DE, Beck JR, Suarez DL, Swayne DE 2006 Susceptibility of North American ducks and gulls to H5N1 highly pathogenic avian influenza viruses. Emerg Infect Dis 12:1663–1670. doi:10.3201/eid1211.060652. 17283615 PMC3372354

[13] Jeong OM, Kim MC, Kim MJ, Kang HM, Kim HR, Kim YJ, Joh SJ, Kwon JH, Lee YJ 2009 Experimental infection of chickens, ducks and quails with the highly pathogenic H5N1 avian influenza virus. J Vet Sci 10:53–60. doi:10.4142/jvs.2009.10.1.53. 19255524 PMC2801098

[14] Hulse-Post DJ, Sturm-Ramirez KM, Humberd J, Seiler P, Govorkova EA, Krauss S, Scholtissek C, Puthavathana P, Buranathai C, Nguyen TD, Long HT, Naipospos TSP, Chen H, Ellis TM, Guan Y, Peiris JSM, Webster RG 2005 Role of domestic ducks in the propagation and biological evolution of highly pathogenic H5N1 influenza viruses in Asia. Proc Natl Acad Sci U S A 102:10682–10687. doi:10.1073/pnas.0504662102. 16030144 PMC1180796

[15] Sims LD, Domenech J, Benigno C, Kahn S, Kamata A, Lubroth J, Martin V, Roeder R 2005 Origin and evolution of highly pathogenic H5N1 avian influenza in Asia. Vet Rec 157:159–164. doi:10.1136/vr.157.6.159. 16085721

[16] Gilbert M, Chaitaweesub P, Parakarnawongsa T, Premashthira S, Tiensin T, Kalpravidh W, Wagner H, Slingenbergh J 2006 Free-grazing ducks and highly pathogenic avian influenza, Thailand. Emerg Infect Dis 12:227–234. doi:10.3201/eid1202.050640. 16494747 PMC3373083

[17] Sturm-Ramirez KM, Hulse-Post DJ, Govorkova EA, Humberd J, Seiler P, Puthavathana P, Buranathai C, Nguyen TD, Chaisingh A, Long HT, Naipospos TSP, Chen H, Ellis TM, Guan Y, Peiris JSM, Webster RG 2005 Are ducks contributing to the endemicity of highly pathogenic H5N1 influenza virus in Asia? J Virol 79:11269–11279. doi:10.1128/JVI.79.17.11269-11279.2005. 16103179 PMC1193583

[18] Perkins LEL, Swayne DE 2001 Pathobiology of A/Chicken/Hong Kong/220/97 (H5N1) avian influenza virus in seven gallinaceous species. Vet Pathol 38:149–164. doi:10.1354/vp.38-2-149. 11280371

[19] Adams SC, Xing Z, Li J, Cardona CJ 2011 Immune-related gene expression in response to H5N1 avian influenza virus infection in chicken and duck embryonic fibroblasts. Mol Immunol 49:413–413. doi:10.1016/j.molimm.2011.10.003. 19250679

[20] Kuchipudi SV, Dunham SP, Nelli R, White GA, Coward VJ, Slomka MJ, Brown IH, Chang KC 2012 Rapid death of duck cells infected with influenza: a potential mechanism for host resistance to H5N1. Immunol Cell Biol 90:116–123. doi:10.1038/icb.2011.17. 21423263 PMC3257048

[21] Liang QL, Luo J, Zhou K, Dong JX, He HX 2011 Immune-related gene expression in response to H5N1 avian influenza virus infection in chicken and duck embryonic fibroblasts. Mol Immunol 48:924–930. doi:10.1016/j.molimm.2010.12.011. 21256597

[22] Mo IP, Brugh M, Fletcher OJ, Rowland GN, Swayne DE 1997 Comparative pathology of chickens experimentally inoculated with avian influenza viruses of low and high pathogenicity. Avian Dis 41:125–136. doi:10.2307/1592452. 9087329

[23] Swayne DE, Beck JR 2005 Experimental study to determine if low-pathogenicity and high-pathogenicity avian influenza viruses can be present in chicken breast and thigh meat following intranasal virus inoculation. Avian Dis 49:81–85. doi:10.1637/7260-081104R. 15839417

[24] Das A, Spackman E, Thomas C, Swayne DE, Suarez DL 2008 Detection of H5N1 high pathogenicity avian influenza virus in meat and tracheal samples from experimentally infected chickens. Avian Dis 52:40–48. doi:10.1637/8093-082107-Reg. 18459294

[25] Tumpey TM, Suarez DL, Perkins LEL, Senne DA, Lee JG, Lee YJ, Mo IP, Sung HW, Swayne DE 2002 Characterization of a highly pathogenic H5N1 avian influenza A virus isolated from duck meat. J Virol 76:6344–6355. doi:10.1128/JVI.76.12.6344-6355.2002. 12021367 PMC136198

[26] Antarasena C, Sirimujalin R, Prommuang P, Blacksell SD, Promkuntod N, Prommuang P 2006 Tissue tropism of a Thailand strain of high-pathogenicity avian influenza virus (H5N1) in tissues of naturally infected native chickens (Gallus gallus), Japanese quail (Coturnix coturnix japonica) and ducks (Anas spp). Avian Pathol 35:250–253. doi:10.1080/03079450600714510. 16753617

[27] Mase M, Eto M, Tanimura N, Imai K, Tsukamoto K, Horimoto T, Kawaoka Y, Yamaguchi S 2005 Isolation of a genotypically unique H5N1 influenza virus from duck meat imported into Japan from China. Virology 339:101–109. doi:10.1016/j.virol.2005.05.010. 15964604

[28] De Rossi M, Bernasconi P, Baggi F, Malefyt RD, Mantegazza R 2000 Cytokines and chemokines are both expressed by human myoblasts: possible relevance for the immune pathogenesis of muscle inflammation. Int Immunol 12:1329–1335. doi:10.1093/intimm/12.9.1329. 10967028

[29] Tournadre A, Miossec P 2007 Cytokine response in inflammatory myopathies. Curr Rheumatol Rep 9:286–290. doi:10.1007/s11926-007-0046-6. 17688837

[30] Nagaraju K, Raben N, Merritt G, Loeffler L, Kirk K, Plotz P 1998 A variety of cytokines and immunologically relevant surface molecules are expressed by normal human skeletal muscle cells under proinflammatory stimuli. Clin Exp Immunol 113:407–414. doi:10.1046/j.1365-2249.1998.00664.x. 9737670 PMC1905062

[31] Baquero-Perez B, Kuchipudi SV, Nelli RK, Chang KC 2012 A simplified but robust method for the isolation of avian and mammalian muscle satellite cells. BMC Cell Biol 13:16. doi:10.1186/1471-2121-13-16. 22720831 PMC3432597

[32] Harper DR 1993 Virology labfax. Blackwell Scientific Publications, Oxford, United Kingdom.

[33] Finney DJ 1971 Probit analysis, 3rd ed Cambridge University Press, London, United Kingdom.

[34] Peters MA, Browning GF, Washington EA, Crabb BS, Kaiser P 2003 Embryonic age influences the capacity for cytokine induction in chicken thymocytes. Immunology 110:358–367. doi:10.1046/j.1365-2567.2003.01744.x. 14632664 PMC1783060

[35] Barber MRW, Aldridge JR, Webster RG, Magor KE 2010 Association of RIG-I with innate immunity of ducks to influenza. Proc Natl Acad Sci U S A 107:5913–5918. doi:10.1073/pnas.1001755107. 20308570 PMC2851864

[36] Karpala AJ, Stewart C, McKay J, Lowenthal JW, Bean AGD 2011 Characterization of chicken MDA5 activity: regulation of IFN-beta in the absence of RIG-I functionality. J Immunol 186:5397–5405. doi:10.4049/jimmunol.1003712. 21444763

[37] Spackman E, Senne DA, Myers TJ, Bulaga LL, Garber LP, Perdue ML, Lohman K, Daum LT, Suarez DL 2002 Development of a real-time reverse transcriptase PCR assay for type A influenza virus and the avian H5 and H7 hemagglutinin subtypes. J Clin Microbiol 40:3256–3260. doi:10.1128/JCM.40.9.3256-3260.2002. 12202562 PMC130722

[38] Kuchipudi SV, Tellabati M, Nelli RK, White GA, Perez BB, Sebastian S, Slomka MJ, Brookes SM, Brown IH, Dunham SP, Chang KC 2012 18S rRNA is a reliable normalisation gene for real time PCR based on influenza virus infected cells. Virol J 9:230. doi:10.1186/1743-422X-9-230. 23043930 PMC3499178

[39] Slomka MJ, Pavlidis T, Coward VJ, Voermans J, Koch G, Hanna A, Banks J, Brown IH 2009 Validated real time reverse transcriptase PCR methods for the diagnosis and pathotyping of Eurasian H7 avian influenza viruses. Influenza Other Respir Viruses 3:151–164. doi:10.1111/j.1750-2659.2009.00083.x. 19627372 PMC4634683

[40] Kuchipudi SV, Nelli R, White GA, Bain M, Chang KC, Dunham SP 2009 Differences in influenza virus receptors in chickens and ducks: implications for interspecies transmission. J Mol Genet Med 3:143–151. doi:10.4172/1747-0862.1000026. 19565022 PMC2702077

[41] McArdle A, Maglara A, Appleton P, Watson AJ, Grierson I, Jackson MJ 1999 Apoptosis in multinucleated skeletal muscle myotubes. Lab Invest 79:1069–1076. 10496525

[42] Turpin SM, Lancaster GI, Darby I, Febbraio MA, Watt MJ 2006 Apoptosis in skeletal muscle myotubes is induced by ceramides and is positively related to insulin resistance. Am J Physiol Endocrinol Metab 291:E1341–E1350. doi:10.1152/ajpendo.00095.2006. 16849630

[43] Liniger M, Summerfield A, Zimmer G, McCullough KC, Ruggli N 2012 Chicken cells sense influenza A virus infection through MDA5 and CARDIF signaling involving LGP2. J Virol 86:705–717. doi:10.1128/JVI.00742-11. 22072756 PMC3255855

[44] O'Neill MC, Kendal AP 1975 Infection of differentiating muscle cells with influenza and Newcastle disease viruses. Nature 253:195–198. doi:10.1038/253195a0. 1167401

[45] Whittaker G, Bui M, Helenius A 1996 Nuclear trafficking of influenza virus ribonucleoproteins in heterokaryons. J Virol 70:2743–2756. 8627748 10.1128/jvi.70.5.2743-2756.1996 PMC190131

[46] Irmler M, Thome M, Hahne M, Schneider P, Hofmann B, Steiner V, Bodmer JL, Schroter M, Burns K, Mattmann C, Rimoldi D, French LE, Tschopp J 1997 Inhibition of death receptor signals by cellular FLIP. Nature 388:190–195. doi:10.1038/40657. 9217161

[47] Koseki T, Inohara N, Chen S, Nunez G 1998 ARC, an inhibitor of apoptosis expressed in skeletal muscle and heart that interacts selectively with caspases. Proc Natl Acad Sci U S A 95:5156–5160. doi:10.1073/pnas.95.9.5156. 9560245 PMC20230

[48] Desdouits M, Munier S, Prevost MC, Jeannin P, Butler-Browne G, Ozden S, Gessain A, Van der Werf S, Naffakh N, Ceccaldi PE 2013 Productive infection of human skeletal muscle cells by pandemic and seasonal influenza A(H1N1) viruses. PLoS One 8:e79628. doi:10.1371/journal.pone.0079628. 24223983 PMC3818236

[49] Vassalli P 1992 The pathophysiology of tumor necrosis factors. Annu Rev Immunol 10:411–452. doi:10.1146/annurev.iy.10.040192.002211. 1590993

[50] Londt BZ, Nunez A, Banks J, Alexander DJ, Russell C, Richard-Londt AC, Brown IH 2010 The effect of age on the pathogenesis of a highly pathogenic avian influenza (HPAI) H5N1 virus in Pekin ducks (Anas platyrhynchos) infected experimentally. Influenza Other Respir Viruses 4:17–25. doi:10.1111/j.1750-2659.2009.00116.x. 20021503 PMC4941950

[51] Ko JH, Jin HK, Asano A, Takada A, Ninomiya A, Kida H, Hokiyama H, Ohara M, Tsuzuki M, Nishibori M, Mizutani M, Watanabe T 2002 Polymorphisms and the differential antiviral activity of the chicken Mx gene. Genome Res 12:595–601. doi:10.1101/gr.210702. 11932243 PMC187515

[52] Benfield CTO, Lyall JW, Kochs G, Tiley LS 2008 Asparagine 631 variants of the chicken Mx protein do not inhibit influenza virus replication in primary chicken embryo fibroblasts or in vitro surrogate assays. J Virol 82:7533–7539. doi:10.1128/JVI.00185-08. 18508886 PMC2493316

[53] Ewald SJ, Kapczynski DR, Livant EJ, Suarez DL, Ralph J, McLeod S, Miller C 2011 Association of Mx1 Asn631 variant alleles with reductions in morbidity, early mortality, viral shedding, and cytokine responses in chickens infected with a highly pathogenic avian influenza virus. Immunogenetics 63:363–375. doi:10.1007/s00251-010-0509-1. 21286706

[54] Li ZJ, Jiang YP, Jiao PR, Wang AQ, Zhao FJ, Tian GB, Wang XJ, Yu KZ, Bu ZG, Chen HL 2006 The NS1 gene contributes to the virulence of H5N1 avian influenza viruses. J Virol 80:11115–11123. doi:10.1128/JVI.00993-06. 16971424 PMC1642184

[55] Liniger M, Moulin HR, Sakoda Y, Ruggli N, Summerfield A 2012 Highly pathogenic avian influenza virus H5N1 controls type I IFN induction in chicken macrophage HD-11 cells: a polygenic trait that involves NS1 and the polymerase complex. Virol J 9:7. doi:10.1186/1743-422X-9-7. 22230322 PMC3283523

[56] Moulin HR, Liniger M, Python S, Guzylack-Piriou L, Ocana-Macchi M, Ruggli N, Summerfield A 2011 High interferon type I responses in the lung, plasma and spleen during highly pathogenic H5N1 infection of chicken. Vet Res 42:6. doi:10.1186/1297-9716-42-6. 21314963 PMC3031227

[57] Koerner I, Kochs G, Kalinke U, Weiss S, Staeheli P 2007 Protective role of beta interferon in host defense against influenza A virus. J Virol 81:2025–2030. doi:10.1128/JVI.01718-06. 17151098 PMC1797552

[58] Toffan A, Beato MS, De Nardi R, Bertoli E, Salviato A, Cattoli G, Terregino C, Capua I 2008 Conventional inactivated bivalent H5/H7 vaccine prevents viral localization in muscles of turkeys infected experimentally with low pathogenic avian influenza and highly pathogenic avian influenza H7N1 isolates. Avian Pathol 37:407–412. doi:10.1080/03079450802061124. 18622857 PMC2562020

[59] Yoneyama M, Kikuchi M, Natsukawa T, Shinobu N, Imaizumi T, Miyagishi M, Taira K, Akira S, Fujita T 2004 The RNA helicase RIG-I has an essential function in double-stranded RNA-induced innate antiviral responses. Nat Immunol 5:730–737. doi:10.1038/ni1087. 15208624

